# Instance causal multiple instance learning for whole slide image classification

**DOI:** 10.1371/journal.pone.0362621

**Published:** 2026-11-11

**Authors:** Haixing Li, Chao Weng, Wanying Huang, Ming Xu

**Affiliations:** 1 Shenyang University of Technology, Shenyang, China; 2 Shengjing Hospital of China Medical University, Shenyang, China; Wroclaw University of Science and Technology, POLAND

## Abstract

Whole slide images (WSIs) are characterized by extremely high resolution and usually lack fine-grained annotations, making multiple instance learning (MIL) the dominant paradigm for weakly supervised WSI classification. Although existing MIL methods have achieved substantial progress in feature extraction and instance aggregation, most of them overlook the influence of confounding factors, thereby learning spurious associations that impair generalization performance. To address this issue, we develop an Instance Causal Multiple Instance Learning (ICMIL) framework for WSI classification, which aims to reduce the model’s reliance on potentially spurious instance-level associations. Specifically, unlike conventional likelihood-based strategies, the proposed method introduces a backdoor-adjustment-inspired prototype module during instance aggregation, where clustered prototypes are used as proxy contextual patterns to mitigate the influence of potential confounding cues. We conduct extensive experiments on two publicly available WSI datasets, Camelyon16 and TCGA-NSCLC. The results demonstrate that the proposed ICMIL framework achieves competitive performance and generally improves existing baselines, while also exhibiting a plug-and-play property.

## Introduction

With the development of digital whole slide scanning technology, histopathology slides can now be digitized into gigapixel whole slide images (WSIs), thereby promoting automated analysis based on computer vision and deep learning [1,2]. In clinical practice, particularly in oncologic pathology, the core task is usually to make a slide-level diagnosis. However, such decisions often rely on only a small number of diagnostically relevant local regions, while these critical pieces of evidence are sparsely distributed across the entire slide and occupy only a tiny fraction of it [3,4]. Meanwhile, available datasets typically provide only slide-level labels without fine-grained region-level annotations [3,5]. Moreover, manual annotation of lesion regions at high resolution is not only extremely costly but also difficult to scale to large datasets [6,7]. Therefore, WSI classification is naturally formulated as a multiple instance learning (MIL) problem, in which each slide is regarded as a bag composed of a large number of image patches [5,8,9].

MIL is a weakly supervised learning paradigm in which each WSI is modeled as a bag, and the thousands of image patches are treated as instances [10]. It is negative only if all instances are negative [11]. In this setting, each bag is associated with a single label, and the model must learn to identify the key instances that determine this label [12]. As a result, MIL has become a common formulation for computer-aided WSI analysis and has demonstrated strong empirical performance in this domain [1,13]. Attention-based MIL methods have become the predominant paradigm for WSI analysis [14]. Their core idea is to learn importance weights for individual instances, thereby enabling the model to focus on key regions during aggregation, improving classification performance and providing instance-level interpretability [8,15,16].

Although existing MIL methods have made significant progress in feature extraction and instance aggregation [9,17], most of them ignore the influence of confounding factors (e.g., staining variations, scanner-induced artifacts, and tissue preparation differences), thereby learning spurious correlations that impair generalization performance [17,18]. For instance, in routine histopathology, staining color often acts as a typical confounding factor. As shown in Fig 1, most patches in positive bags are stained purple, whereas patches in negative bags are stained pink. When patches of certain colors frequently co-occur with bag labels, the model may be misled into relying on patch color information for bag-level prediction [19–21]. Therefore, a reliable and interpretable MIL model should establish an effective mechanism to reduce reliance on spurious correlations induced by potential confounding or contextual factors and encourage aggregation based on label-relevant histopathological evidence, thereby improving the performance and generalization ability of MIL methods [9,18,22].

**Fig 1:**
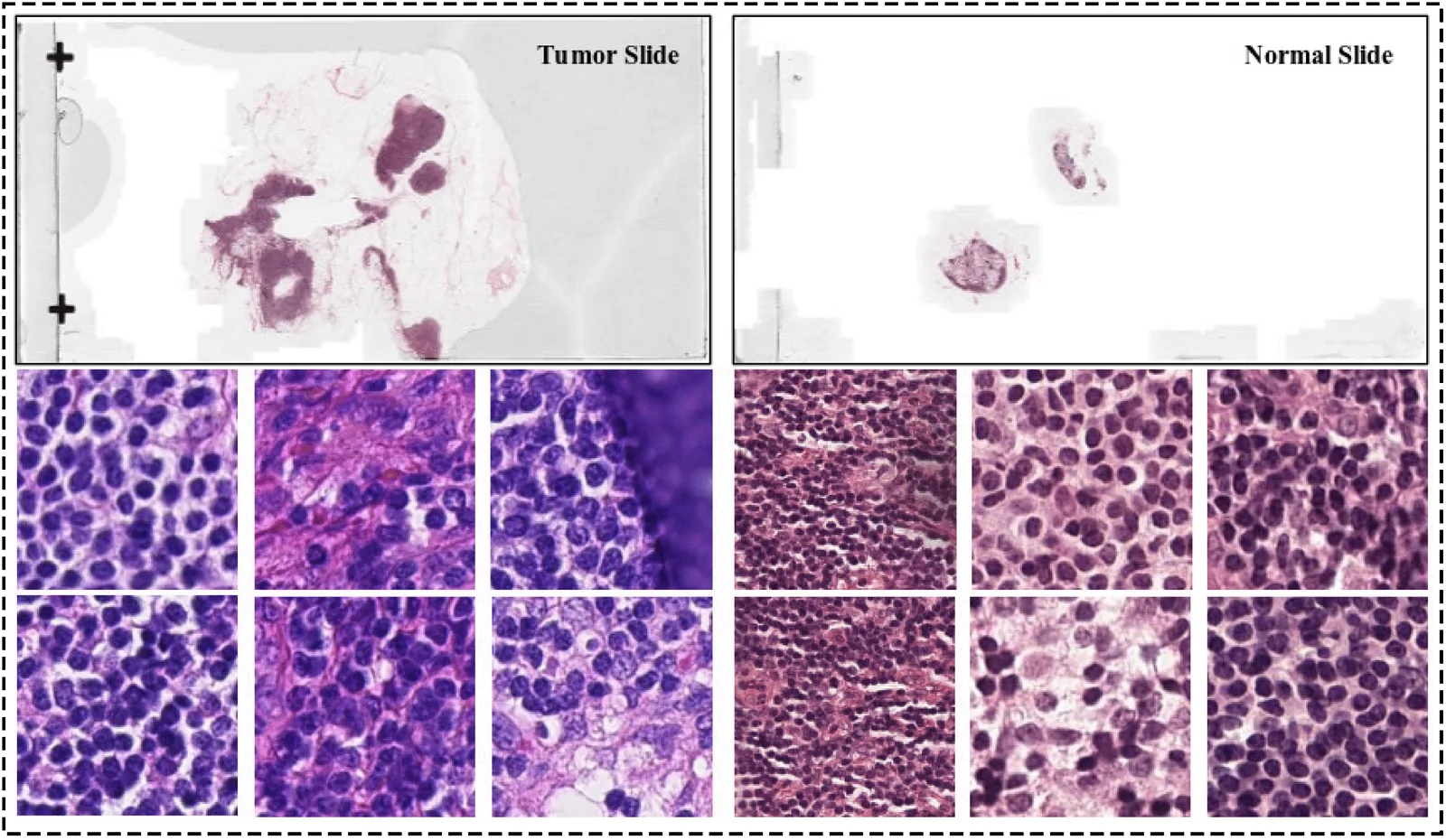
Confounding factors illustration. In patches from WSIs, positive patches are stained purple, whereas negative patches are stained pink. When specific color features frequently co-occur with class labels, the model may be misled into relying on color information for bag-level prediction.

In fact, learning robust and less biased bag-level representations is highly challenging [23,24], because spurious associations are often latent and difficult to observe, yet they can substantially undermine the performance of MIL methods [24–26]. To address this issue, we propose an Instance Causal Multiple Instance Learning (ICMIL) method for WSI classification. Since true confounding factors are usually latent and not fully annotated, we approximate recurrent patch-level contextual patterns using a prototype dictionary and employ a backdoor-adjustment-inspired formulation to mitigate their potential influence during instance aggregation.

## Materials and methods

### Dataset and preprocessing

We evaluated our proposed method on two publicly available WSI datasets: Camelyon16 and TCGA-NSCLC. For both datasets, we adopted the standard preprocessing pipeline proposed in CLAM [3]. Specifically, we removed background and non-tissue regions, and subsequently divided the valid tissue regions of each WSI into non-overlapping patches of 256 × 256 pixels.

Camelyon16 is a WSI dataset for breast cancer metastasis detection, consisting of 399 WSIs. Using the official split, which is strictly partitioned at the patient level, we adopted 270 WSIs for training and 129 WSIs for testing. Model performance was evaluated on the test set.

TCGA-NSCLC contains two non-small cell lung cancer subtypes: lung adenocarcinoma (LUAD) and lung squamous cell carcinoma (LUSC). This dataset includes 541 LUAD WSIs and 512 LUSC WSIs, totaling 1053 WSIs from 956 cases. Using the official split, which is strictly partitioned at the patient (case) level to prevent data leakage, we utilized 843 WSIs for training and 210 WSIs for testing.

Following the standard feature extraction pipeline established by CLAM [3], we used a ResNet50 model initialized with ImageNet pretrained weights to extract patch-level features [10,16,27,28]. Specifically, after standard preprocessing (i.e., color normalization using ImageNet statistics), each image patch was fed into the pretrained ResNet50 backbone. Consistent with the CLAM configuration, we extracted the representations after the third residual block followed by spatial pooling, yielding a 1024-dimensional feature vector (*d* = 1024) for each corresponding patch.Using an ImageNet-pretrained encoder allows the model to leverage rich visual representations learned from large-scale natural images, which has been shown to improve feature quality and training stability in histopathological image analysis [10,29]. To mitigate the massive memory footprint associated with WSI processing, the weights of the ResNet50 feature extractor were kept frozen, meaning no fine-tuning was performed during this stage. The extracted patch features were subsequently used as input to the downstream MIL aggregation and classification modules.

### Methods

#### MIL formulation.

Within the MIL framework, a WSI is treated as a bag containing *N* patches, denoted by $S=\{s_{i}{\}}_{i=1}^{N}$, where $s_{i}$ is the *i*-th patch. The instance-level label $y_{i}\in \{0,1\}$ is latent and unavailable during training. Under the standard MIL assumption, the bag label *Y* is defined as


$$\begin{array}{c} Y=\left\{ \begin{array}{cc} 0, & \text{iff }\sum_{i=1}^{N}y_{i}=0,\\ 1, & \text{otherwise}. \end{array}\right. \end{array}$$ (1)

An attention-based MIL model typically consists of three stages: (1) instance feature extraction via a feature extractor f(·), (2) attention-based aggregation σ(·), (3) bag-level prediction with a classifier g(·). The process can be formulated as


$$\begin{array}{cc} x_{i} & =f(s_{i}),\\ B & =\sigma (\{x_{i}{\}}_{i=1}^{N}),\\ \hat{Y} & =g(B), \end{array}$$ (2)

where $s_{i}$ is the *i*-th patch, $x_{i}$ denotes its feature embedding extracted by the feature extractor f(·), σ(·) is the aggregation function that combines all instance features into a bag-level representation *B*, and g(·) is the bag-level classifier that outputs the predicted slide label $\hat{Y}$.

#### Causal inference in MIL.

We formulate the MIL framework as a causal graph with three variables: instance features I, bag feature B, and confounding factors C, as shown in Fig 2A. Here, B denotes the aggregated bag representation learned from multiple instances rather than the bag label itself. The directed edges describe the following relationships.

I→B: This represents the causal effect of instance features on the bag feature. The bag feature B is aggregated from multiple instance features I, characterizing the primary instance-to-bag predictive relationship.
C→I: This indicates the effect of the confounding factor on instance features. In real clinical workflows, WSIs acquired in practice are often influenced by confounding factors, which may introduce bias.
C→B: This indicates that the instance aggregation process is affected by the confounding factors. A MIL model may incorrectly assign high importance to purple-stained patches when such color patterns are spuriously correlated with slide-level labels.

**Fig 2:**
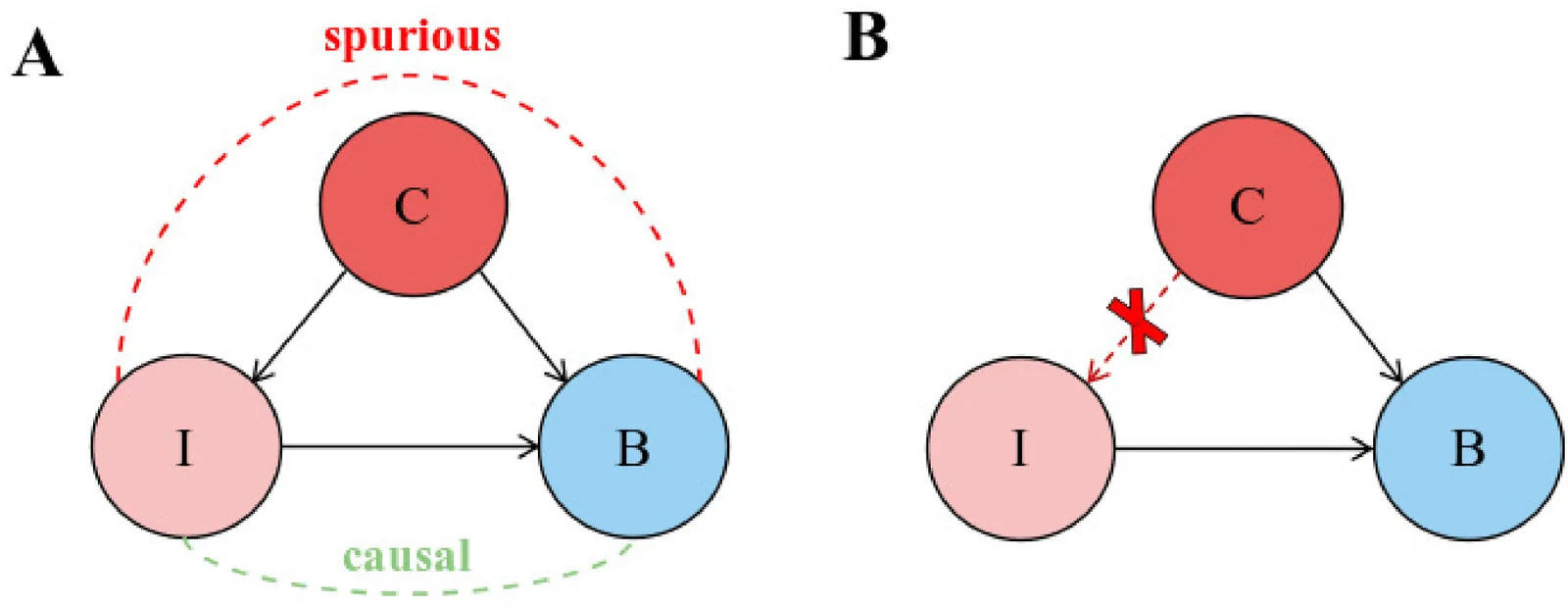
The causal graph for MIL. (A) Causal graph. (B) P(B∣do(I)).

The confounding factor *C* confounds *I* and *B* through the backdoor path I←C→B, creating a spurious correlation between them. This hinders the instance aggregation process and consequently degrades the performance and generalization ability of the MIL model. Ideally, An MIL model should prioritize label-relevant instance-to-bag associations. However, the conventional likelihood-based formulation P(B∣I) may be insufficient to account for biases associated with latent contextual factors. To address this issue, we instead consider the interventional distribution P(B∣do(I)), where do(·) denotes forcibly assigning a specific value to variable *I*. As illustrated in Fig 2B, this operation can be interpreted as an intervention on the original causal graph, blocking the backdoor path between *I* and *B* and thereby mitigating the effect of confounding. In theory, the ideal way to perform such an intervention is through randomized controlled trials, which would require exhaustively enumerating each bag under all possible contextual conditions. However, this is impractical in real-world applications. Therefore, we propose a practical intervention strategy to reduce the confounding effect introduced by the confounding factor.

#### Instance causal multiple instance learning.

We propose to use the backdoor adjustment formula to realize ICMIL, P(B∣do(I)), for instance aggregation. The backdoor adjustment is given as follows:


$$\begin{array}{c} P(B|do(I))=\sum_{k=1}^{K}P(B|I,h(I,c_{k}))P(c_{k}), \end{array}$$ (3)

where $c_{k}$ denotes each element in the confounding factor set, and h(·) is a mapping function to be determined (see Eq. (4)). In this formulation, $P(c_{k})$ represents the prior contribution of each prototype. In the absence of prior knowledge about the prevalence of different prototypes, we use a uniform prior. This provides a practical approximation for incorporating prototype-level contextual information during instance aggregation, with the goal of reducing reliance on potentially confounding visual patterns. Fig 3 illustrates overview of the proposed ICMIL framework.

**Fig 3:**
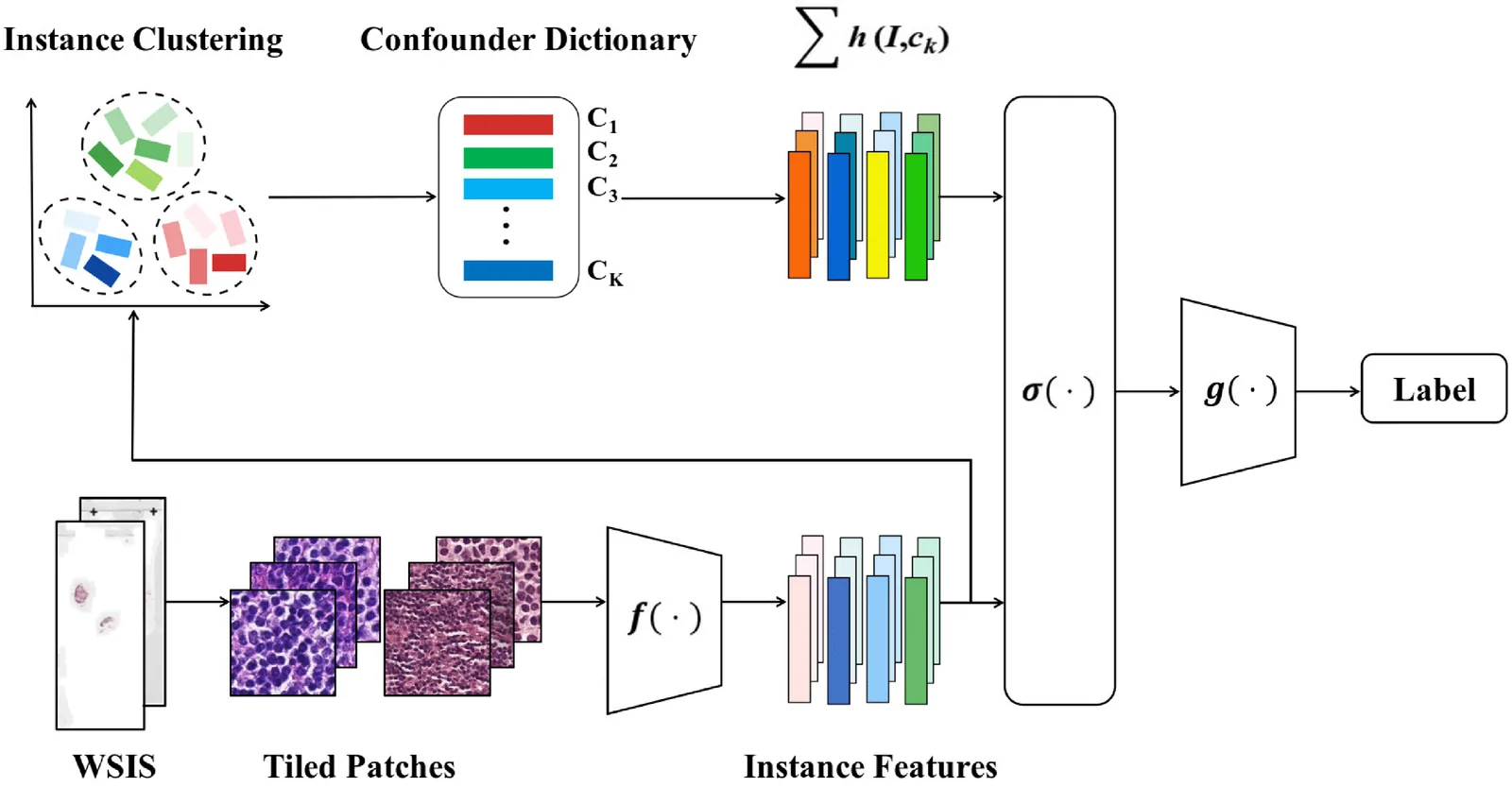
Overview of the proposed ICMIL framework. A WSI is first divided into multiple patches for instance feature extraction, while instance clustering is used to construct a proxy confounding factor dictionary. The instance features are then adjusted through intervention, followed by MIL aggregation and classification for slide-level prediction.

Due to the difficulty of explicitly enumerating all potential confounding factors, we construct the confounding factor dictionary as a set of prototype vectors extracted in an unsupervised manner from the feature distribution of the training set. Specifically, during dictionary construction, we first load a preliminarily trained MIL model, pool the instance features from all training bags, and perform K-means clustering on the pooled features. The centroid of each cluster is regarded as a proxy prototype for potential confounding or contextual patterns, and all centroids together constitute the confounding factor dictionary, denoted by $𝐂=[{𝐜}_{1},...,{𝐜}_{K}]\in {\mathbb{R}}^{d\times K}$.

It should be noted that these prototypes are not assumed to be directly observed ground-truth confounding factors. In WSI classification, true confounding factors, such as staining variation, scanner-related artifacts, tissue preparation differences, background regions, and tissue boundary effects, are usually latent and difficult to annotate explicitly. Therefore, the clustered prototypes are used as a compact approximation of recurrent patch-level patterns in the training feature space. These recurrent patterns may influence both instance representations and bag-level aggregation, and thus can serve as empirical proxies for potential confounding factors in the backdoor adjustment. Meanwhile, because the dictionary is constructed without semantic supervision, some prototypes may also contain disease-relevant histological structures. Thus, the confounding factor dictionary should be interpreted as an approximate representation of latent confounding or contextual patterns rather than a definitive set of true causal confounding factors. Next, we define:


$$\begin{array}{cc} h(I,c_{k}) & =\left[h(s_{1},c_{k}),...,h(s_{N},c_{k})\right],\\ h(s_{j},c_{k}) & =\alpha_{jk}c_{k},\\ \left[\alpha_{j1},...,\alpha_{jK}\right] & =\mathrm{softmax}\;\left(\frac{\left(W_{q}s_{j}{)}^{⊤}(W_{k}C\right)}{\sqrt{d_{a}}}\right), \end{array}$$ (4)

where $s_{j}\in {\mathbb{R}}^{d}$ denotes the feature of the *j*-th instance, $c_{k}\in {\mathbb{R}}^{d}$ denotes the *k*-th instance-level confounding factor prototype. $W_{q},W_{k}\in {\mathbb{R}}^{d_{a}\times d}$ are learnable projection matrices used to map the instance features and confounding factor prototypes into a shared attention space. $\sqrt{d_{a}}$ is a scaling factor, where $d_{a}$ denotes the dimension of the shared attention space. We further define:


$$\begin{array}{c} P(B|I,h(I,c_{k}))=P(B|I⊕h(I,c_{k})), \end{array}$$ (5)

where ⊕ denotes vector concatenation. In the absence of prior knowledge about the prevalence of different confounding factor prototypes, we assume a uniform prior $P(c_{k})=1/K$, so that no prototype is preferentially weighted during intervention. In principle, Eq. (3) requires *K* forward passes to enumerate all confounding factor prototypes. To reduce the computational cost, we approximate the expectation over $P(c_{k})$ by aggregating the confounding factor-aware representations before bag-level prediction:


$$\begin{array}{c} P(B|do(I))\approx P\left(B|I⊕\sum_{k=1}^{K}h(I,c_{k})P(c_{k})\right). \end{array}$$ (6)

Thus, backdoor adjustment can be achieved by one feed forward of the network.

## Results

### Performance comparison with existing works

We mainly compared our method with ABMIL, DSMIL, TransMIL, and DTFD-MIL, all of which are attention-based MIL approaches. In addition, two conventional MIL pooling strategies, max pooling and mean pooling, were also included for comparison. All models were implemented using PyTorch and trained on a single NVIDIA RTX 3090 GPU. The MIL classifier was optimized using the Adam optimizer with a batch size of 1, an initial learning rate of $1\times 10^{-4}$, and a weight decay of $1\times 10^{-4}$. The learning rate was scheduled using a cosine annealing strategy, with a minimum learning rate of $5\times 10^{-6}$. The standard Cross-Entropy loss was utilized as the objective function. We trained the models for a maximum of 200 epochs and employed an early stopping mechanism with a patience of 20 epochs to prevent overfitting. For the proposed ICMIL, the size of the confounding factor dictionary was empirically set to *K* = 16 in all main experiments. To ensure robust evaluation and mitigate the variance caused by network initialization, all experiments were conducted over five independent runs with different random seeds.

As shown in Tables 1 and 2, compared with attention-based MIL methods, Mean-pooling and Max-pooling achieved inferior results, which may be mainly attributed to the low proportion of positive instances in Camelyon16, typically less than 10%. In such a setting, mean pooling is easily dominated by abundant negative instances, whereas max pooling cannot effectively exploit the global information of all instances within a bag. Compared with the original MIL methods, ICMIL achieves performance improvements to varying degrees on both datasets, indicating that the proposed method can effectively reduce the influence of confounding information during bag-level aggregation. As a result, the model is encouraged to focus more on key instances that are directly related to category discrimination, rather than being distracted by background noise or spuriously correlated features. The superior results on Camelyon16 suggest that the discriminative cues in this dataset are more concentrated and less affected by confounding factors. In contrast, TCGA-NSCLC contains more complex histopathological variations and task-irrelevant information, which are more likely to form spurious correlations with the labels, thereby increasing the difficulty of identifying key instances.

**Table 1:** The performance of different MIL approaches on Camelyon16. Results are presented as mean ± SD.

Model	Acc	F1	AUC
Mean-pooling	77.71±1.54	70.13±1.37	79.92±1.48
Max-pooling	79.64±1.42	72.66±1.38	84.26±1.79
DTFD-MIL	91.00±1.41	89.10±1.10	94.10±0.82
ICMIL(DTFD-MIL)	92.53±1.26	90.72±1.27	95.56±1.29
DSMIL	89.68±1.44	87.11±1.24	94.09±1.33
ICMIL(DSMIL)	93.44±1.16	91.37±1.31	95.83±0.88
TransMIL	90.77±0.94	87.17±1.32	95.01±1.08
ICMIL(TransMIL)	91.83±1.12	88.74±1.10	96.62±1.29
ABMIL	90.44±1.11	89.77±0.92	96.15±1.18
ICMIL(ABMIL)	94.00±1.19	92.22±1.06	97.18±1.03

**Table 2:** The performance of different MIL approaches on TCGA-NSCLC. Results are presented as mean ± SD.

Model	Acc	F1	AUC
Mean-pooling	82.33±1.70	80.01±1.37	85.93±1.84
Max-pooling	82.59±1.42	82.18±1.31	85.46±1.21
DTFD-MIL	88.04±1.38	87.94±1.10	93.08±1.44
ICMIL(DTFD-MIL)	90.60±1.20	88.85±1.18	95.94±1.08
DSMIL	89.58±0.93	86.31±0.91	94.38±1.49
ICMIL(DSMIL)	90.36±0.83	88.65±1.43	95.04±1.28
TransMIL	89.42±1.17	89.32±1.53	96.52±1.29
ICMIL(TransMIL)	90.52±1.32	89.46±1.34	97.13±1.12
ABMIL	90.61±0.94	89.01±1.40	95.71±1.53
ICMIL(ABMIL)	91.71±0.88	90.72±1.04	97.81±0.90

### Comparison with representative causal MIL methods

To further evaluate the effectiveness of the proposed ICMIL, we compare it with three representative causal MIL methods for WSI classification, including CATTMIL, CaMIL, and IBMIL. These methods introduce causal reasoning into MIL from different perspectives. CATTMIL mainly improves the attention mechanism to reduce the influence of spuriously attended instances. CaMIL incorporates causal modeling into the MIL framework to enhance robustness under weak supervision. IBMIL performs interventional learning at the bag level to alleviate the confounding effect caused by bag contextual priors. For a fair comparison, all methods are implemented based on the same ABMIL backbone. As shown in Table 3, ICMIL achieves the best performance on both Camelyon16 and TCGA-NSCLC across all evaluation metrics. Specifically, on Camelyon16, ICMIL achieves 94.00% accuracy, 92.22% F1, and 97.18% AUC, outperforming the second-best model (CaMIL) by 1.90%, 1.47%, and 0.89%, respectively. On TCGA-NSCLC, ICMIL also obtains the highest accuracy, F1, and AUC, reaching 91.71%, 90.72%, and 97.81%, respectively. These results indicate that ICMIL consistently outperforms existing causal MIL methods under the same backbone.

**Table 3:** The comparison with representative causal MIL methods on Camelyon16 and TCGA-NSCLC.

Model	Camelyon16			TCGA-NSCLC		
	Acc	F1	AUC	Acc	F1	AUC
IBMIL(ABMIL)	90.86	88.24	93.19	89.73	87.52	92.17
CATTMIL(ABMIL)	91.33	89.36	95.14	90.66	88.12	94.07
CaMIL(ABMIL)	92.10	90.75	96.29	91.23	89.18	94.55
ICMIL(ABMIL)	94.00	92.22	97.18	91.71	90.72	97.81

The superior performance of ICMIL can be mainly attributed to its instance-level causal intervention. Unlike IBMIL, which performs intervention on bag-level representations, ICMIL introduces backdoor adjustment during the instance aggregation stage. Therefore, confounding information can be suppressed before the final bag representation is formed. This is particularly suitable for WSI classification, because confounding factors such as staining variation, background regions, and tissue preparation artifacts usually appear at the patch level. By constructing an instance-level confounding factor dictionary, ICMIL can more directly reduce patch-level biases and encourage the model to focus on truly discriminative histopathological regions, leading to more reliable bag representations and better classification performance.

### Ablation on model design variants

#### Size of confounding factor dictionary.

We conducted ablation experiments on the size of the confounding factor dictionary *K* on two datasets, including Camelyon16 and TCGA-NSCLC. As shown in Fig 4, ICMIL maintains strong performance under different choices of *K* on both Camelyon16 and TCGA-NSCLC, demonstrating the effectiveness and robustness of the proposed method. Overall, a moderate dictionary size achieves the best results, suggesting that this scale is sufficient to capture representative confounding patterns and thus enables effective backdoor adjustment.

**Fig 4:**
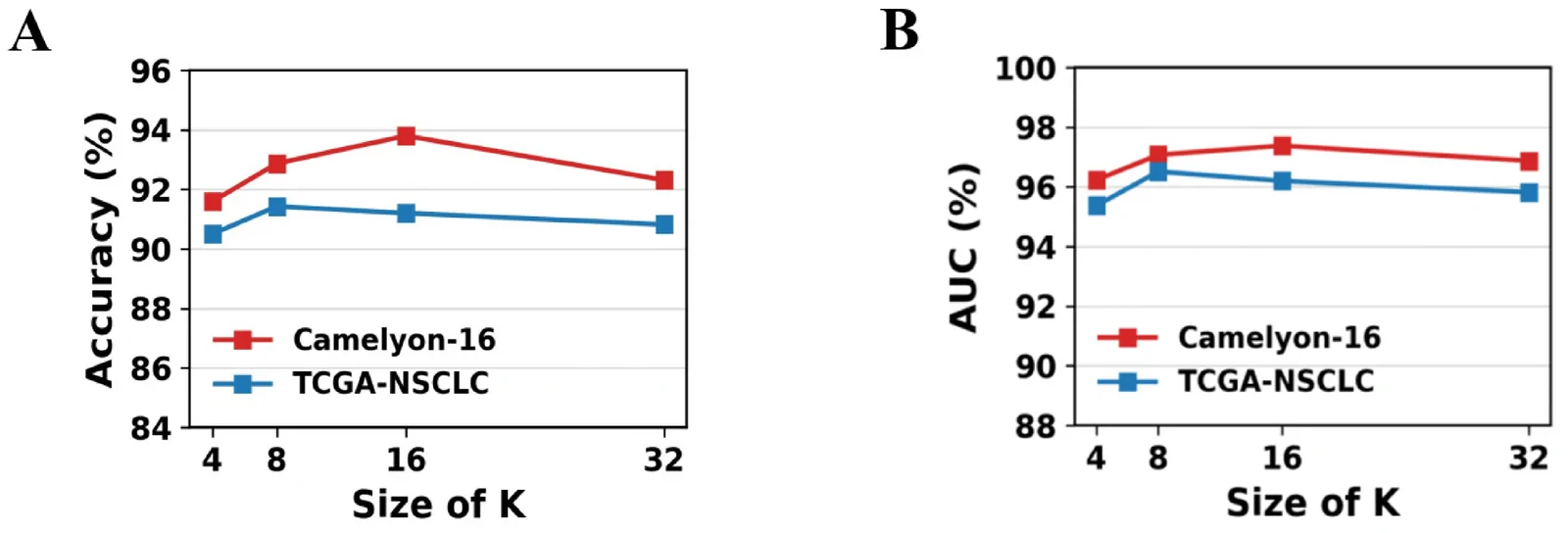
Ablation study on the size of the confounding factor dictionary *K.* Performance of ICMIL with different dictionary size on Camelyon16 and TCGA-NSCLC in terms of (A) Accuracy. (B) AUC.

#### Implementation of backdoor adjustment.

To investigate how confounding factors should be integrated into the original instance features, we compared three implementations of backdoor adjustment at the instance level, where ⊕ denotes vector concatenation, + denotes element-wise addition, and – denotes element-wise subtraction. As shown in Fig 5, all three implementations led to performance improvements, demonstrating the robustness and effectiveness of the proposed ICMIL method.

**Fig 5:**
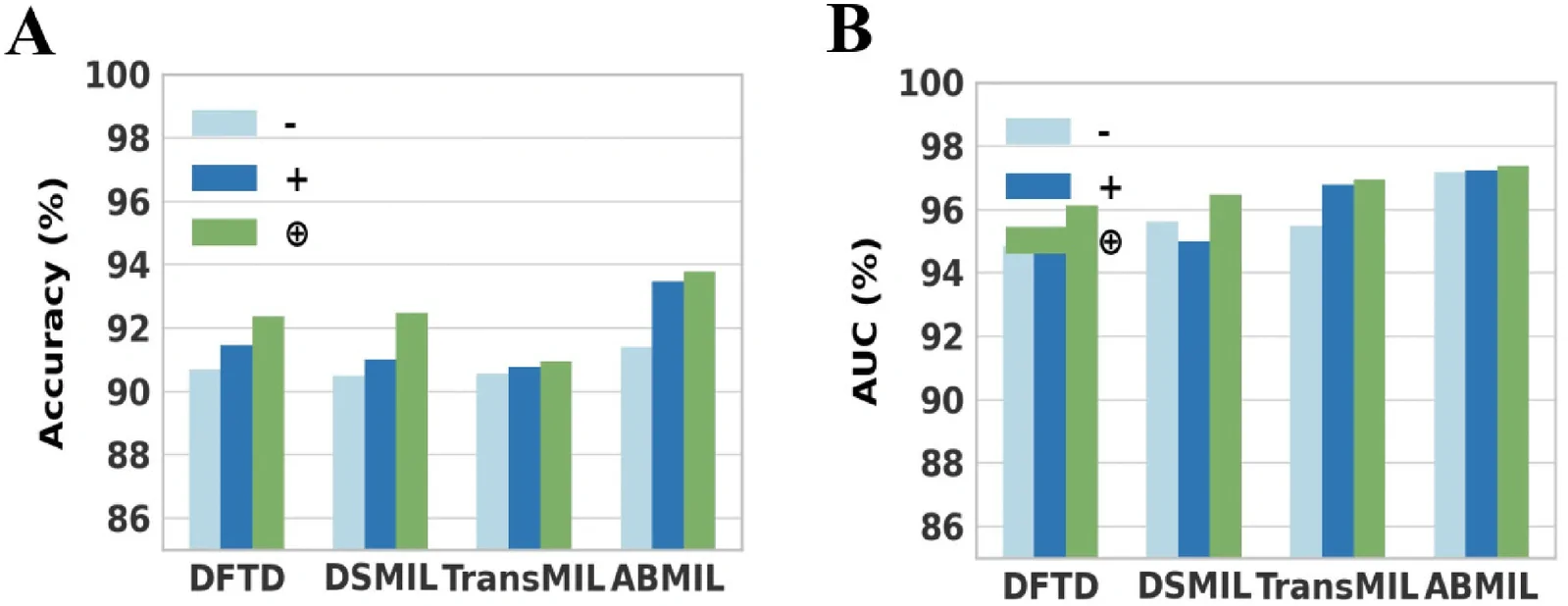
Ablation study on implementations of backdoor adjustment. Performance of ICMIL with different implementation of backdoor adjustment in terms of (A) Accuracy. (B) AUC.

#### Analysis of the impact of color normalization.

To investigate the influence of stain preprocessing, we applied color normalization to the WSIs before feature extraction. As shown in Table 4, color normalization provided modest improvements for ABMIL on both datasets. More importantly, ICMIL consistently outperformed the corresponding ABMIL baseline with and without color normalization. Under the normalized setting, ICMIL improved all three metrics on both datasets, including accuracy gains of 1.02 and 0.51 percentage points on CAMELYON-16 and TCGA-NSCLC, respectively.

**Table 4:** The performance comparison of models with and without color normalization.

Model	Camelyon16			TCGA-NSCLC		
	Acc	F1	AUC	Acc	F1	AUC
ABMIL	90.44	89.77	96.15	90.61	89.01	95.71
ABMIL-Norm	90.53	90.04	96.85	90.72	89.22	95.78
ICMIL(ABMIL-Norm)	91.55	91.20	96.89	91.23	90.18	96.55
ICMIL(ABMIL)	94.00	92.22	97.18	91.71	90.72	97.81

ICMIL without color normalization achieved the best overall performance, reaching 94.00% accuracy and 97.18% AUC on CAMELYON-16, and 91.71% accuracy and 97.81% AUC on TCGA-NSCLC. These results indicate that the effectiveness of ICMIL does not depend on explicit color normalization. A possible explanation is that color normalization may remove subtle pathological information, while ICMIL can reduce reliance on potentially confounding contextual patterns during feature aggregation without additional stain preprocessing.

### Visual analysis of model interpretability

One key aspect of clinical translation is the deep integration of such tools into routine pathology workflows. We envision the multiple instance learning approach as serving primarily as an assistive tool rather than replacing pathologists, functioning as a digital second reader or a triage support system in the diagnostic process. In addition, attention heatmaps provide a more intuitive basis for interpreting the predictions by highlighting the regions on which the model focuses during decision-making.

As shown in Fig 6, the original H&E slide was first subjected to tissue region segmentation to remove the interference from large background areas, thereby enabling the model to focus on the actual tissue structures. The subsequently generated interpretability heatmap reflected the relative contribution of different spatial regions to the model’s classification decision, in which purple regions indicated areas assigned higher importance or attention by the model, suggesting that these regions played a more critical role in the final prediction. In contrast, yellow regions indicated areas of lower importance, implying a relatively limited contribution to the classification outcome.

**Fig 6:**
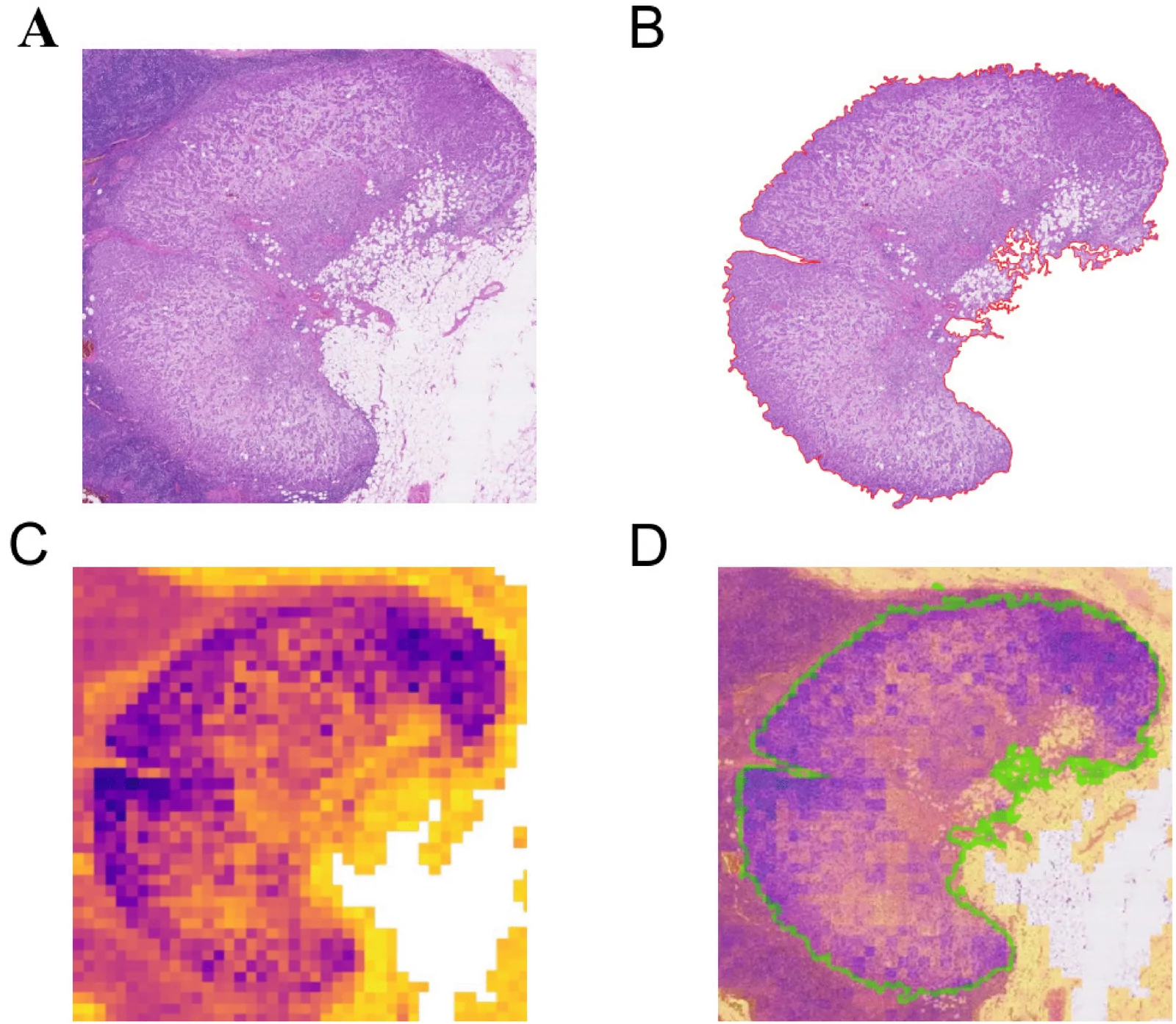
Visual explanation of the attention mechanism for model interpretability. (A) H&E Slide. (B) Tissue Segmentation Mask. (C) Interpretability Heatmap. (D) Heatmap Overlay Example.

The model’s high-response regions are mainly concentrated within the tissue parenchyma and show good agreement with the valid regions identified by tissue segmentation, rather than being distributed in non-tissue areas such as the background, blank regions, or slide boundaries. This suggests that, after introducing the confounding factor-aware adjustment, the model primarily focuses on morphologically informative tissue regions, such as parenchymal areas with dense cellular or nuclear structures, rather than non-biological or weakly informative regions such as blank background, slide borders, tissue edges, or obvious staining.

## Discussion

In this study, we proposed ICMIL, an instance causal multiple instance learning framework for WSI classification. ICMIL incorporates a backdoor-adjustment-inspired module into the instance aggregation stage. Our results suggest that incorporating prototype-level contextual adjustment may improve the robustness of slide-level prediction by mitigating reliance on potential confounding or contextual cues. Compared with conventional likelihood-based MIL methods, our approach is designed to encourage greater attention to histopathologically meaningful instances and reduce reliance on spuriously correlated cues.

The performance gains observed on Camelyon16 and TCGA-NSCLC support this interpretation. In routine H&E WSIs, color distribution, background patterns, and other acquisition-related factors may co-occur with class labels and thus bias the aggregation process. By incorporating a confounding factor dictionary and performing backdoor adjustment during feature aggregation, ICMIL aims to weaken these spurious associations and encourage the model to capture more label-relevant histopathological evidence. This may explain why ICMIL achieved overall improvements over several representative MIL baselines.

We also observed that the magnitude of improvement varied across datasets and backbone models. A possible explanation is that the discriminative cues in Camelyon16 are relatively concentrated, whereas TCGA-NSCLC contains more complex histomorphological variation and more task-irrelevant information. Under such conditions, spurious correlations may be harder to remove completely, and the benefit of causal intervention may depend on the aggregation mechanism of the underlying MIL architecture. Therefore, our findings suggest that ICMIL is a promising and generally effective framework, although its compatibility and gain may vary across different MIL backbones and application settings.

Another important aspect of this study is interpretability. The attention heatmaps showed that high-response regions were mainly distributed within tissue parenchyma rather than in background regions, blank areas, or slide boundaries. This observation indicates that the model predictions are less likely to be driven by obvious non-biological artifacts and are more likely to rely on histologically meaningful structures. From a clinical perspective, this property is important for trust and usability, and it supports the potential role of ICMIL as an assistive tool, such as a digital second reader or a triage support system, rather than a replacement for pathologists.

This study has several limitations. First, the proposed method was evaluated on two public datasets only, and its generalizability to multi-center clinical data remains to be established. Second, the confounding factor dictionary was constructed in an unsupervised manner using prototype clustering, which may not fully characterize the true distribution of confounding factors in WSI data. Third, the prior over confounding factors was simplified as a uniform distribution, which may limit the precision of backdoor adjustment. In addition, this study focused on slide-level classification, and the value of the proposed framework for broader pathology tasks still requires further investigation.

In future work, we will explore more efficient strategies for confounding factor approximation and replace the uniform prior with data-driven conditional priors. External validation on larger and more diverse cohorts will also be necessary to assess robustness under different staining conditions, scanners, and clinical workflows. These efforts may further improve the practical value of causal intervention for computational pathology.

## Conclusion

We propose an instance causal multiple instance learning framework for WSI classification, termed ICMIL, which aims to mitigate the influence of potential confounding or contextual patterns during instance aggregation. Rather than relying solely on conventional likelihood-based modeling, ICMIL incorporates a prototype dictionary constructed from training-set instance features as proxy contextual variables in a backdoor-adjustment-inspired formulation. Experiments on two benchmark WSI datasets suggest that ICMIL generally improves several representative MIL baselines. Future work will focus on more accurate strategies for approximating latent contextual factors and replacing the uniform prior with data-driven conditional priors.
