# Supplementary material for: Instance causal multiple instance learning for whole slide image classification
Source: PLoS One. 2026 Nov 11;21(11):e0362621. doi: 10.1371/journal.pone.0362621 (PMC13754216; doi:10.1371/journal.pone.0362621)
Supplement: Supplementary file 1 [file pone.0362621.s002.docx]

Response to Reviewer #1

Manuscript: Instance Causal Multiple Instance Learning for

Whole Slide Image Classification

Dear Reviewer,

We sincerely thank you for your careful reading and constructive comments. We have revised the manuscript substantially to clarify the causal interpretation, correct and expand the dataset description, improve implementation transparency, report multi-run results, add comparisons with related causal MIL methods, and moderate our claims. Our point-by-point responses are provided below.

1. Interpretation of cluster centroids/prototypes as confounders

Reviewer comment: Could the authors explain why the cluster centroids/prototypes can be interpreted as confounders? Since the confounder dictionary is constructed using unsupervised clustering of instance features, it is unclear whether these representative values capture true confounding factors or disease-relevant histological patterns. The authors should also clarify what WSI features these clusters represent and what features the model primarily focuses on during prediction.

Response:

Thank you for raising this important conceptual issue. We agree that an unsupervised cluster centroid cannot be identified as a ground-truth causal confounder solely because it is obtained by K-means clustering. We have therefore revised the manuscript to avoid this strong interpretation. In ICMIL, the centroids are treated as proxy prototypes of recurrent patch-level contextual patterns in the training feature space, not as directly observed or semantically pure confounders.

The rationale is that latent acquisition- and preparation-related factors—such as staining variation, scanner artifacts, tissue preparation differences, background patterns, and tissue-boundary effects—alter patch representations and may also influence the attention-based aggregation used to form the bag representation. Recurrent feature-space prototypes can compactly summarize such patterns and can therefore be used as empirical proxy variables in a backdoor-adjustment-inspired module. However, because dictionary construction is unsupervised, some prototypes may also encode disease-relevant morphology. We now state this limitation explicitly and describe the dictionary as an approximate representation of latent confounding or contextual patterns rather than a definitive set of true causal confounders.

Regarding what the model focuses on, the revised qualitative analysis indicates that high-attention regions are concentrated in tissue parenchyma, particularly regions with dense cellular or nuclear structures, rather than blank background, slide borders, tissue edges, or obvious staining artifacts. We have correspondingly limited the interpretability claim to the spatial attention evidence shown; we do not claim that each prototype has a unique pathological meaning without additional prototype-level annotation.

Location of revision: Changes in the manuscript: causal motivation and proxy interpretation, pp. 2 and 5 (lines 40–44 and 128–147); attention-map description, pp. 9–10 (lines 249–270 and 298–305); limitation of unsupervised dictionary construction, p. 10 (lines 306–310).

2. TCGA-NSCLC composition and split

Reviewer comment: Please revisit the dataset description, especially for the TCGA-NSCLC dataset. The reported number of LUAD and LUSC WSIs does not appear to match the reported training/test split. The authors should correct this discrepancy and clarify whether the split was performed at the patient/case level or slide level.

Response:

Thank you for identifying the ambiguity in the earlier description. We rechecked the dataset counts and revised this section. TCGA-NSCLC contains 541 LUAD WSIs and 512 LUSC WSIs, totaling 1,053 WSIs from 956 cases. The revised split contains 843 training WSIs and 210 test WSIs; these numbers sum to 1,053.

Most importantly, the partition was performed strictly at the patient/case level, not at the slide level. All WSIs belonging to the same TCGA case were assigned to only one partition, thereby preventing patient-level information leakage. We have stated this explicitly in the revised manuscript.

Location of revision: Changes in the manuscript: Dataset and Preprocessing, p. 3, lines 47–60.

3. Implementation details and reproducibility

Reviewer comment: Could the authors provide more detailed implementation information to improve reproducibility? Important details such as preprocessing parameters, patch extraction settings, training epochs, validation strategy, random seeds, hyperparameter settings, and confounder dictionary construction should be described more clearly. Providing a GitHub repository or source code would substantially improve reproducibility.

Response:

We agree and have expanded the implementation description. Both datasets follow the CLAM preprocessing pipeline: background/non-tissue regions are removed; valid tissue regions are divided into non-overlapping 256 × 256-pixel patches; patches are normalized with ImageNet statistics; and a frozen ImageNet-pretrained ResNet50 is used for feature extraction. Features are taken after the third residual block and spatial pooling, producing a 1,024-dimensional vector per patch.

The MIL classifier is trained in PyTorch on one NVIDIA RTX 3090 GPU using Adam, batch size 1, initial learning rate 1 × 10⁻⁴, weight decay 1 × 10⁻⁴, cosine annealing with minimum learning rate 5 × 10⁻⁶, and cross-entropy loss. Training lasts at most 200 epochs, with early stopping patience of 20 epochs. The main experiments use K = 16 prototypes and five independent runs.

For dictionary construction, a preliminarily trained MIL model is loaded, training-set instance features are pooled, K-means clustering is applied to those features, and the K centroids form the dictionary. The dictionary is constructed from the training partition only. A uniform prototype prior P(cₖ) = 1/K is used, and the prototype-conditioned representations are aggregated before bag-level prediction to allow a single forward pass.

Location of revision: Changes in the manuscript: preprocessing and feature extraction, p. 3, lines 47–74; dictionary construction and adjustment, pp. 5–6, lines 121–160; optimization and training, p. 6, lines 164–176.

4. Additional dataset and multi-seed robustness

Reviewer comment: Could the authors provide additional experiments to further support the reproducibility and robustness of the proposed method? Evaluation on an additional independent dataset would strengthen the manuscript. In addition, the authors should report mean and standard deviation across multiple random seeds rather than only single-run performance values.

Response:

Thank you for this suggestion. We have repeated all main experiments over five independent runs with different random seeds and now report mean ± standard deviation in Tables 1 and 2. This change provides a more reliable estimate of variability than the previous single-run reporting. We have also included dictionary-size and implementation-variant ablations on both datasets.

We agree that validation on a third independent, preferably multi-center, cohort would provide stronger evidence of external generalizability. Such an experiment is not included in the supplied revision, and we do not wish to imply otherwise. We have therefore acknowledged this limitation explicitly and moderated the generalization claims. External validation under different staining conditions, scanners, and clinical workflows is identified as an important direction for future work.

Location of revision: Changes in the manuscript: five-run protocol, p. 6, lines 174–176; mean ± SD in Tables 1–2, pp. 7–8; robustness ablations, pp. 8–9; external-validation limitation, pp. 10–11, lines 306–317.

5. ICMIL with TransMIL on TCGA-NSCLC and strength of claims

Reviewer comment: In Table 1, ICMIL applied to TransMIL appears to reduce performance on the TCGA-NSCLC dataset compared with the original TransMIL, particularly in terms of AUC. Could the authors explain this result? If the proposed method does not consistently improve performance across all backbones and datasets, the authors should use terms such as ‘superior performance’ or ‘significant improvement’ more carefully.

Response:

We appreciate this careful observation. In the revised manuscript, the TCGA-NSCLC results are reported in Table 2 as mean ± standard deviation over five runs. Under this protocol, ICMIL(TransMIL) does not reduce the mean AUC: TransMIL obtains 89.42 ± 1.17 accuracy, 89.32 ± 1.53 F1, and 96.52 ± 1.29 AUC, whereas ICMIL(TransMIL) obtains 90.52 ± 1.32 accuracy, 89.46 ± 1.34 F1, and 97.13 ± 1.12 AUC. The corresponding mean changes are +1.10, +0.14, and +0.61 percentage points, respectively. The reviewer’s observation likely reflects the earlier single-run table, which has now been replaced by the multi-run analysis.

Nevertheless, we agree with the broader point: the improvement is modest for TransMIL, especially for F1, and the benefit of the prototype adjustment varies with dataset complexity and the aggregation mechanism of the backbone. TCGA-NSCLC contains more heterogeneous morphology and task-irrelevant variation than Camelyon16, which may make spurious correlations harder to remove. We have therefore revised the abstract, discussion, and conclusion to use ‘competitive,’ ‘generally improves,’ and ‘may improve,’ rather than implying a uniform or statistically significant improvement. Because no formal significance test is reported, we do not use ‘significant improvement’ in the statistical sense.

Location of revision: Changes in the manuscript: revised multi-run TCGA-NSCLC results, Table 2; qualified backbone-dependent interpretation, p. 10, lines 289–297; moderated wording in the Abstract and Conclusion, pp. 1 and 11.

6. Comparisons with related causal/interventional MIL methods

Reviewer comment: Could the authors provide comparison experiments with closely related causal or interventional MIL methods, such as Interventional Bag MIL, CaMIL, and Causal Attention MIL, which are cited in the manuscript? Since the main contribution of this study is a causal MIL framework, comparison with these methods would better demonstrate the specific advantage of ICMIL.

Response:

Thank you for this valuable suggestion. We added comparisons with Interventional Bag MIL (IBMIL), Causal Multiple Instance Learning (CaMIL), and Causal Attention MIL (CATTMIL). For a controlled comparison, all methods use the same ABMIL backbone.

As reported in the new Table 3, ICMIL(ABMIL) achieves 94.00 accuracy, 92.22 F1, and 97.18 AUC on Camelyon16, and 91.71 accuracy, 90.72 F1, and 97.81 AUC on TCGA-NSCLC. These are the highest values among the compared causal MIL methods under the shared backbone. We further clarified the methodological distinction: IBMIL intervenes on bag-level representations, whereas ICMIL applies the adjustment during instance aggregation, before the final bag representation is formed; this design directly targets patch-level contextual biases such as staining, background, and preparation artifacts.

Location of revision: Changes in the manuscript: new subsection and Table 3, pp. 7–8, lines 193–218.

7. Pathological validity of attention maps

Reviewer comment: Could the authors provide stronger evidence that the attention maps focus on the expected diagnostic regions of WSIs? The current qualitative heatmap is helpful, but it does not clearly demonstrate whether the model attends to pathologically meaningful regions. Comparison with tumor annotations, pathologist-reviewed regions, or representative diagnostic areas would strengthen the interpretability claim.

Response:

We agree that attention visualization alone does not establish pathological validity and that overlap with tumor annotations or pathologist-reviewed regions would provide substantially stronger evidence. In the supplied revision, we expanded the qualitative description to show that high-response regions are mainly located in tissue parenchyma and dense cellular/nuclear regions, rather than blank background, slide boundaries, or obvious non-biological areas.

However, the current analysis does not include a quantitative comparison with tumor masks or independent pathologist review. We have therefore narrowed our claim: Figure 6 demonstrates that predictions are less likely to be driven by obvious background artifacts, but it does not prove that every highlighted region is diagnostically correct. This limitation should be stated explicitly. A rigorous extension would compare attention with available Camelyon16 tumor annotations using localization metrics and include blinded pathologist assessment of representative high-attention patches.

Location of revision: Changes in the manuscript: qualitative heatmap analysis, pp. 9–10, lines 249–270 and 298–305.

8. Editorial corrections and terminology

Reviewer comment: Minor editorial edits are needed. (a) Line 136: ‘exiting’ should be corrected to ‘existing.’ (b) Please use consistent terminology throughout the manuscript, for example, ‘CAMELYON-16’ and ‘Camelyon-16’ should be standardized to a single form.

Response:

Thank you. We corrected ‘exiting’ to ‘existing’ and carefully proofread the manuscript for related typographical and grammatical issues. We also standardized the dataset name throughout. The supplied PDF consistently uses ‘Camelyon16’; we will retain that single form in the final manuscript and ensure that the abstract, main text, tables, figure captions, and references are checked consistently.

Location of revision: Changes in the manuscript: typographical correction at the location corresponding to the reviewer’s original line 136; terminology standardized throughout the manuscript.

Response to Reviewer #2

Manuscript: Instance Causal Multiple Instance Learning for

Whole Slide Image Classification

Dear Reviewer,

We sincerely thank you for your detailed and constructive comments. We have clarified the dataset composition and preprocessing, expanded the description of the compared MIL architectures and evaluation metrics, explained the use of concatenation, and corrected the editorial inconsistencies. Our point-by-point responses are provided below.

Comment 1

“CAMELYON-16 is variously described in literature as 399 or 400. Please clarify this discrepancy.”

Response

Thank you for identifying this ambiguity. The original CAMELYON-16 challenge webpage described a corpus of 400 WSIs, comprising 270 training slides and 130 test slides. The benchmark version used in this study contains 399 unique/evaluable WSIs (270 training and 129 testing). The difference arises because the duplicated test slide test_049 was removed from the released/evaluable benchmark. We used the 399-slide version and the corresponding split throughout our experiments.

Comment 2

“The actual dataset contains blurred, poorly stained slides, treatment-related artifacts, and questionable positivity slides. If you corrected for these issues by excluding problematic slides, can you improve the performance?”

Response

We agree that CAMELYON-16 contains quality and annotation issues. In the present study, we did not manually exclude slides on the basis of blur, staining quality, treatment-related change, or uncertain positivity. Apart from using the 399-slide benchmark version described above, preprocessing only removed background and non-tissue regions before extracting non-overlapping patches. Therefore, the performance reported in Tables 1–4 reflects the standard benchmark cohort and cannot be interpreted as performance after pathology-led slide-level quality correction.

We retained the standard cohort to preserve direct comparability with prior MIL studies and to avoid creating an easier, selectively filtered test set. We agree that a sensitivity analysis on a pathology-reviewed cleaned cohort would be valuable; however, such an analysis would constitute an additional dataset experiment and should be reported separately with explicit exclusion criteria, the identifiers and labels of excluded slides, the resulting train/test counts, and all metrics recalculated over the same random seeds. We have added this point as a limitation rather than claiming an unperformed performance improvement.

Comment 3

“Given that this data set has extensive staining variability which is inherent in H&Es, was color normalization applied? If not why?”

Response

We thank the reviewer for this important question. The main experiments did not use explicit H&E stain normalization. Before the ImageNet-pretrained ResNet50 encoder, patches underwent only channel-wise normalization using the ImageNet mean and standard deviation; this input normalization should not be confused with histology-specific stain normalization.

To directly evaluate the effect of staining variability, we added the analysis reported in Table 4. For CAMELYON-16, stain normalization changed ABMIL from 90.44% accuracy, 89.77% F1, and 96.15% AUC to 90.53%, 90.04%, and 96.85%, respectively. ICMIL with normalized inputs achieved 91.55% accuracy, 91.20% F1, and 96.89% AUC, whereas ICMIL without explicit stain normalization achieved the best overall CAMELYON-16 result: 94.00% accuracy, 92.22% F1, and 97.18% AUC.

These results show that explicit stain normalization is not required for the effectiveness of ICMIL. A plausible explanation is that aggressive normalization may suppress subtle diagnostically useful color information, whereas the proposed prototype-based adjustment reduces reliance on recurrent contextual patterns during aggregation without requiring a fixed stain template. We have moderated this interpretation because the experiment demonstrates empirical independence from the tested normalization procedure, rather than complete invariance to all possible staining shifts.

Comment 4

“What was the architecture of the MIL being compared?”

Response

We agree that the baseline architectures should be stated explicitly. All embedding-based MIL methods received the same frozen 1024-dimensional patch features extracted after the third residual block and spatial pooling of an ImageNet-pretrained ResNet50. They differed only in their aggregation/classification architecture:

Mean pooling and max pooling: parameter-free pooling across instances, followed by a bag-level classifier.

ABMIL: learned gated-attention weights are used to form an attention-weighted bag representation, followed by a slide-level classifier.

DSMIL: a dual-stream design combines an instance-classification stream with a bag-classification stream centered on the most discriminative instance and its relationships to the remaining instances.

TransMIL: a Transformer-based aggregator models correlations among instances with self-attention and positional information before slide-level classification.

DTFD-MIL: a double-tier feature-distillation architecture partitions a WSI into pseudo-bags, distills discriminative representations in the first tier, and aggregates them for final slide-level prediction in the second tier.

ICMIL is a plug-and-play module placed between patch-feature extraction and the corresponding MIL aggregator. For the comparison with causal MIL methods in Table 3, IBMIL, CATTMIL, CaMIL, and ICMIL were all implemented with the same ABMIL backbone to ensure a controlled comparison.

Comment 5

“Same questions on the TCGA-NSCLC non-small cell lung cancer (NSCLC) dataset.”

Response

We have clarified the same points for TCGA-NSCLC. The cohort used in the manuscript contains 1,053 WSIs from 956 cases: 541 LUAD WSIs and 512 LUSC WSIs. The patient-level split contains 843 training and 210 test WSIs. We did not perform additional manual exclusion for blur, staining quality, artifacts, or questionable subtype labels; tissue segmentation removed background and non-tissue regions. The same frozen ResNet50 feature extractor and the same MIL architectures described above were used.

The main TCGA-NSCLC experiments likewise did not use explicit H&E stain normalization. Table 4 shows that normalization changed ABMIL from 90.61% accuracy, 89.01% F1, and 95.71% AUC to 90.72%, 89.22%, and 95.78%, respectively. ICMIL with normalized inputs achieved 91.23% accuracy, 90.18% F1, and 96.55% AUC, whereas ICMIL without explicit stain normalization achieved 91.71% accuracy, 90.72% F1, and 97.81% AUC.

Because TCGA slides were collected across multiple institutions and scanners, “scan magnification” should not be represented as a single uniform acquisition value unless this was verified from slide metadata. The revised manuscript should report the standardized extraction magnification or microns-per-pixel actually used by the pipeline and, if relevant, describe how slides with different native resolution were resampled.

Comment 6

“The Method section is excellent and complete (mathematically correct). It might be helpful to a reader to briefly explain why concatenation (⊕) is chosen rather than summation.”

Response

Thank you for this helpful suggestion. Concatenation was selected because it preserves the original instance feature and the prototype-aware adjustment as two distinct information streams. The downstream projection/aggregation layer can therefore learn their relative contributions. In contrast, element-wise addition or subtraction requires the two vectors to be dimensionally and semantically aligned and immediately mixes their values; this can cause destructive interference or cancellation and removes information about which component came from the original instance and which came from the adjustment.

The empirical comparison in Fig. 5 is consistent with this rationale: concatenation gives the strongest overall accuracy and AUC across the tested DTFD-MIL, DSMIL, TransMIL, and ABMIL integrations, while addition and subtraction still show that the benefit is not tied exclusively to one fusion operator.

Comment 7

“‘exiting works’ → should be ‘existing works.’”

Response

Thank you. The typographical error has been corrected. The subsection title now reads “Performance comparison with existing works.”

Comment 8

“You may want to explicitly state the evaluation metrics (AUC, accuracy, F1) for the reader.”

Response

We agree and have clarified that performance was evaluated using classification accuracy, the F1 score, and the area under the receiver operating characteristic curve (AUC). Tables 1 and 2 report these metrics as mean ± standard deviation across five independent runs with different random seeds; Tables 3 and 4 report the corresponding comparison values.

Comment 9

“‘maintains strong performance’ is fine, but could be more specific (e.g., stable AUC across K).”

Response

We agree. Fig. 4 evaluates dictionary sizes $K=4,8,16,32$. Both datasets retain high AUC over this range, with only modest variation, showing that performance is not highly sensitive to a single value of $K$. A moderate dictionary size ($K=16$) provides the best overall trade-off and was therefore used in the main experiments.

Comment 10

“Please explicitly state why the results support robustness (e.g., because improvements persist regardless of integration strategy).”

Response

We agree and have made the basis for the robustness claim explicit. The benefit of prototype-aware adjustment persisted when the contextual representation was incorporated through concatenation, element-wise addition, or element-wise subtraction, and the pattern was observed across four different MIL backbones (DTFD-MIL, DSMIL, TransMIL, and ABMIL). Thus, the improvement is not dependent on one specific fusion operator or one specific MIL aggregator. Concatenation nevertheless produced the strongest overall results, which supports its use in the main implementation.
